# Advances in Biosimilars: A Systematic Review of Machine Learning Applications

**DOI:** 10.3390/ph19050745

**Published:** 2026-05-08

**Authors:** Vannessa Duarte, Tomas Gabriel Bas

**Affiliations:** Escuela de Ciencias Empresariales, Universidad Católica del Norte, Larrondo 1281, Coquimbo 1780000, Chile; vannessa.duarte@ucn.cl

**Keywords:** biosimilars, artificial intelligence, machine learning, pharmaceutical development

## Abstract

**Background/Objectives:** Biosimilars are medicinal products derived from reference biologics and designed to demonstrate a high degree of similarity in quality, efficacy, safety, and immunogenicity. Machine learning (ML) and other artificial intelligence (AI) methodologies have emerged as important tools in this field in biosimilar research and development. This systematic review identifies ML applications throughout the biosimilar lifecycle while distinguishing them from the broader AI literature and from health technology evaluation, economic, and decision-analytic studies. **Methods:** Following PRISMA, records were retrieved from Scopus, PubMed, and Web of Science. After applying predefined inclusion and exclusion criteria, 44 original peer-reviewed studies were selected. Only studies that implemented a data-driven ML method for a biosimilar-relevant problem were included. **Results:** The review mapped AI applications at different stages of biosimilar development and characterized emerging trends and the types of methods used at each stage. Evidence indicates that the most mature empirical ML applications are concentrated in manufacturing optimization and analytical comparability, where supervised learning, ensemble models, and neural networks support process control, glycan or spectral analysis, and similarity assessment. By contrast, biosimilar-specific ML applications in clinical prediction and pharmacovigilance remain comparatively limited. **Conclusions:** These advances support the mission of biosimilars to provide affordable and high-quality biologic therapies. Using ML, developers can reduce timelines, reduce costs, and strengthen safety and efficacy assessments through the analysis of complex datasets that are difficult to address with traditional approaches. The main contribution of this review is to provide a clearer map of methodological maturity, translational relevance, and future opportunities for data-driven biosimilar development.

## 1. Introduction

Biosimilars are active pharmaceutical substances produced from original biological drugs, formulated with a high degree of similarity in terms of quality, efficacy, safety, and immunogenicity compared to reference drugs [1,2]. Unlike generic drugs, which have small molecules, biosimilars are not exact replicas, but rather large complex proteins synthesized in living organisms, which can lead to minute variations from the original [3]. Therefore, demonstrating biosimilarity requires comparative precision assessments to determine the absence of clinically significant differences with respect to the reference product [4,5]. It is essential to recognize that even small differences in manufacturing processes can generate variations that could affect the chemical structure and biological activity of biosimilars in patients [6,7]. However, these variations, although minimal, highlight the importance of vigilance throughout the entire production cycle, including after marketing, through continuous monitoring to address any emerging safety issues related to immunogenicity or treatment efficacy [8,9,10,11]. This development and regulatory approval process is rigorous and costly, and for this purpose, frameworks have been established by internationally renowned agencies such as the European Medicines Agency (EMA) and the Food and Drug Administration (FDA) responsible for ensuring that similarity to reference biological products is demonstrated [2,12,13].

Currently, ML and various AI methodologies have become established as cutting-edge tools in the field of pharmaceutical research and development. ML algorithms demonstrate an exceptional ability to identify patterns in large and complex datasets, making them particularly suitable for analyzing high-dimensional analytical data and optimizing multifactor manufacturing processes in biosimilar development. Some specific applications of ML in protein engineering include optimizing protein structures and predicting functional outcomes, which significantly improves therapeutic efficacy [14]. In process optimization, ML algorithms streamline bioprocesses, improving production yield and efficiency, and thus reducing operating costs [15]. Techniques such as deep learning and generative models have transformed the drug discovery landscape by allowing the rapid identification of potential drug candidates and the personalization of therapies [16,17]. Furthermore, quality control is strengthened through predictive modeling, where ML facilitates real-time monitoring of product attributes, ensuring consistency and compliance with safety standards [18,19]. However, the implementation of neural network algorithms and decision trees also facilitates the path to the commercialization of biosimilars, clearly indicating how these ML algorithms improve productivity while simultaneously driving innovation throughout the production chain [19,20,21,22,23].

Similarly, the literature has identified the analytical potential of classification techniques, such as Random Forests (RF), Support Vector Machines (SVM), and Artificial Neural Networks (ANN), which improve the precision of drug characterization and clinical outcome prediction [24]. Furthermore, studies focused on model optimization through feature selection highlight the importance of customized approaches in predictive modeling, crucial for effectively managing the regulatory environments of the biopharmaceutical industry [25]. Using large datasets, ML facilitates the analytical characterization of biosimilars, providing information that offers comparisons of effectiveness with reference biologics [26]. All of these advances position ML as an important tool in current biosimilar market strategies [27]. This innovation potential reflects a larger shift in the pharmaceutical industry towards data-driven decision-making, innovating in biosimilar development practices [28,29].

This article is a systematic review of primary empirical studies, rather than an original experimental investigation. Consequently, the unit of analysis is the published study, and the core synthesis is restricted to articles that implemented a data-driven machine-learning approach on a biosimilar-specific problem. To avoid conceptual inflation, broader studies related to AI, symbolic or rule-based computational approaches, and HTA/economic decision models were not treated as empirical evidence of ML unless they included an actual trained learning component. The objective of this review is therefore to identify where genuine ML applications have been demonstrated across the biosimilar lifecycle, to distinguish them from adjacent computational literature, and to determine which stages show relatively mature evidence versus those that remain exploratory.

## 2. Methods

### 2.1. Search Strategy

The study was conducted using three databases: Scopus; PubMed, and Web of Science (WoS). Scopus was chosen for its robust and extensive indexing of relevant journals in biosimilars, biotechnology, and data science, as well as its efficient export capabilities for analysis, making it the preferred database for literature searches in these fields [30,31]. WoS and PubMed were considered even though many of the articles retrieved from WoS and PubMed were already included in the Scopus database. The search was carried out in late September 2025 and was not restricted by publication date, language, or journal, except for the criteria indicated below. A comprehensive query was conducted that combined ML keywords with terms related to biosimilars and various phases of drug development. In summary, the search used similar terms across both databases. Phrases such as “Machine Learning” OR “Artificial Intelligence” OR “deep learning” were used. Database-specific terms strategy was designed to include any study that applied ML techniques to problems in the development, production, characterization, or evaluation of biosimilars (Table 1).

### 2.2. Selection of Articles for Analysis of the Results

**Figure 1:**
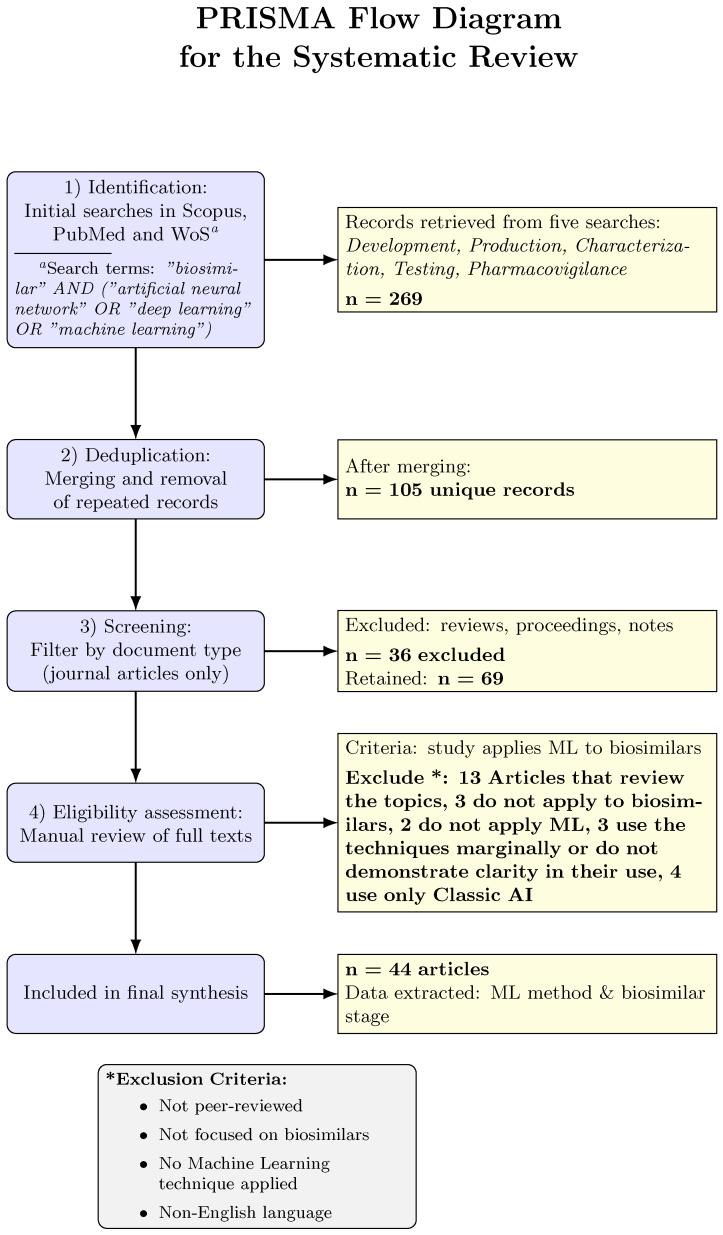
Methodology used to conduct the bibliographic review.

We follow the PRISMA guidelines for study selection (Figure 1). After conducting the search in Scopus, PubMedMethods and WoS, all retrieved records were merged based on their DOI, retaining only the first occurrence, and duplicates were removed. Subsequently, all articles whose document type differed from “Article” were excluded. We applied the inclusion and exclusion criteria in two selection phases: first, a review of titles and abstracts, and then a review of the full text as follows:

- Inclusion criteria: (1) Studies must be articles in peer-reviewed journals presenting original research (not reviews or editorials [32,33] or notes [3,34,35]); (2) the study focuses on biosimilars (development, production, analysis, or use of biosimilar products), and (3) the study employs at least one ML or AI technique as part of its methodology or analysis.
- Exclusion criteria: Articles not written in English were excluded (to ensure that the methodology was interpreted), as were studies not focused on biosimilars (e.g., general bioprocessing studies without a biosimilar context) and studies that did not apply any ML techniques (e.g., if only mentioned conceptually). Conference proceedings, theses [36,37], and other unpublished works were also excluded to maintain consistency [38,39,40,41]. In particular, review articles were excluded to focus on empirical research [1,19,42,43,44,45,46,47,48,49,50,51,52,53,54,55,56,57,58,59,60,61,62,63]; if a review was found in the search results, it was discarded rather than included in the analysis of ML applications.

Two reviewers (the authors) initially screened titles and abstracts to exclude clearly irrelevant records (e.g., review on the topic [64,65,66,67,68,69,70,71,72,73,74,75,76] or sole opinions or discussions about policies or approved medications [77,78,79]). The full text of the remaining articles was then obtained to assess their eligibility in detail. At this stage, any article that only marginally mentioned ML [80,81] or that did not clearly demonstrate its application in the context of biosimilars (e.g., articles that cited an algorithm but did not implement it or where the connection to biosimilar development was tangential) [82,83,84] were excluded.

Disagreements about inclusion were resolved through discussion and a final list of included studies were agreed upon.

After the selection process, a total of 44 articles met all the criteria and were included in our review (see Table A1). The initial literature search retrieved 269 records in various queries targeting different stages of biosimilars. After merging the results and removing duplicates, 105 unique records remained. We then examined these 105 records, excluding 36 records that did not meet our inclusion criteria (this group consisted mainly of review articles, conference papers, notes, or articles unrelated to biosimilars or lacking any ML component). This selection left 69 research articles for detailed evaluation. We read the full text of these 69 articles and excluded another 25 for insufficient relevance; in most cases, those excluded at this stage mentioned ML only tangentially or, upon closer examination, did not specifically apply ML to a biosimilar problem. Finally, 44 studies were confirmed to directly applied ML methods in the context of biosimilar development or analysis and were included in the final analysis. This selection process follows a PRISMA-style workflow (Figure 1) (identification: 269 records; selection: 69; eligibility assessment: 44 included (Table A1)).

### 2.3. Operational Definition of Empirical ML

For the purposes of this review, empirical ML was defined as the training, validation, optimization, or implementation of a data-driven algorithm that learns patterns from biosimilar-relevant data in order to support prediction, classification, clustering, similarity assessment, process optimization, or pharmacovigilance signal detection. This definition includes supervised learning, unsupervised learning, ensemble methods, deep learning, and data-driven optimization approaches when fitted to empirical biosimilar datasets. To improve conceptual precision, the evidence base was classified into two tiers.

Tier 1: Empirical evidence for core ML. These studies met all inclusion criteria and formed the basis for the main synthesis.

Tier 2: Contextual computational literature. These studies included broader AI-related papers, symbolic or rule-based approaches, and HTA/economic/decision analysis frameworks that were relevant to the wider biosimilar ecosystem but did not constitute empirical evidence of ML for the purposes of the main synthesis. These studies were retained only for contextual discussion and, where appropriate, additional reporting.

In this framework, Markov [85,86] or semi-Markov models [87], microsimulation, budget-impact analysis, and similar decision-analytic approaches [88] were not classified as ML unless they explicitly incorporated a trained learning algorithm.

### 2.4. Data Extraction and Analysis

For each study included in the core empirical ML synthesis, we extracted the year of publication, journal, biosimilar context, dataset type, ML method, task performed, and biosimilar lifecycle stage addressed. Lifecycle stages were grouped into five categories:

1. Development and design: early-stage development including molecular design, in silico studies, and formulation development.
2. Production and manufacturing: development of bioprocesses, optimization of manufacturing (upstream and downstream processes), and scaling up.
3. Characterization and quality control: analytical characterization, comparability exercises, and quality assurance tests to establish similarity to the reference product.
4. Clinical trials (efficacy and safety): preclinical studies and clinical trials that evaluate the efficacy, safety, and immunogenicity of the biosimilar.
5. Pharmacovigilance and post-marketing surveillance: post-approval surveillance of the safety and efficacy of biosimilars, including real-world evidence and monitoring of adverse events.

Each included study was assigned to one or more stages in the lifecycle according to its main analytical contribution. The synthesis was primarily descriptive and interpretive. We summarized the distribution of empirical ML applications across stages, identified the most frequently used ML approaches, and qualitatively assessed where the evidence base appears to be more mature versus more exploratory. Contextual computational studies identified during the screening were not included in the main counts or figures.

## 3. Results

### 3.1. Brief Summary of the Articles Studied

**Figure 2:**
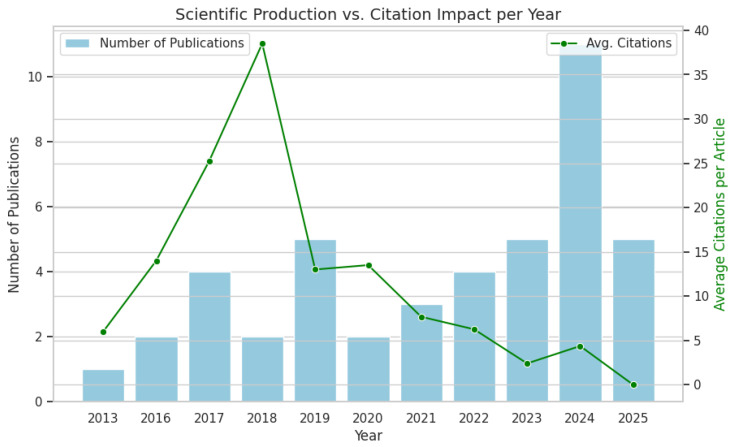
Distribution of publications on biosimilars and ML over the years and impact of citation.

The 44 included studies were published between 2013 and 2025, showing a clear evolution in both volume and scientific impact (Figure 2). No relevant articles were found prior to 2013, marking it as the starting point for ML applications in biosimilar research. Although the mid-2010s saw only sporadic publications, a significant upward trend emerged in the late 2010s and early 2020s, reflecting a broader adoption of data-driven methods in pharmaceutical research. Although the count for 2025 remains incomplete due to the search cutoff in September, the ongoing activity underscores a sustained momentum in the field.

This growth in publication volume is contrasted by the citation dynamics (represented by the green line in Figure 2). As expected, the average impact of the citation is greater for previous studies, which have had more time to solidify their influence. The citation distribution particularly highlights the end-2010s as a pivotal period, for instance, the work by Sokolov et al. (2018) [89], who introduced a hybrid model for production and characterization. In contrast, more recent articles from 2020 onward show lower citation counts, a typical pattern reflecting the natural delay in academic recognition. In general, this combined trend illustrates a maturing research niche in which pioneering studies from the 2010s established the foundation for the current expansion of AI in biologic and biosimilar development.

**Figure 3:**
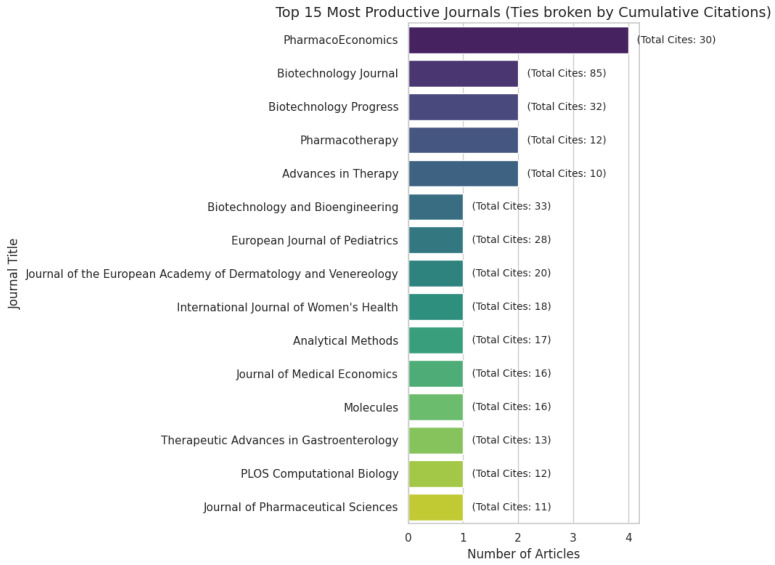
Top 15 articles distributed by journal and cites.

The journals highlighted in Figure 3 represent the top 15 sources in terms of included articles. This distribution underscores the multidisciplinary nature of research at the intersection of biosimilars and ML, with the 44 selected studies spanning a wide range of scientific fields. *PharmacoEconomics* represented the largest share of publications, followed by a group of highly represented journals, including the *Biotechnology Journal*, *Journal of Pharmaceutical Sciences*, *Biotechnology Progress*, *Journal of Medical Economics*, *Pharmacotherapy* and *Advances in Therapy*. Together, these venues span disciplines ranging from biotechnology and engineering to clinical therapeutics and health economics.

The refined analysis indicates that the journal distribution is prominently centered on biotechnology, pharmaceutical sciences, process engineering, and analytical characterization. This shift reinforces the conclusion that the most established empirical ML applications in biosimilars are in the technical and comparability-oriented stages of product development.

### 3.2. AI Techniques Used in Biosimilar Research

**Figure 4:**
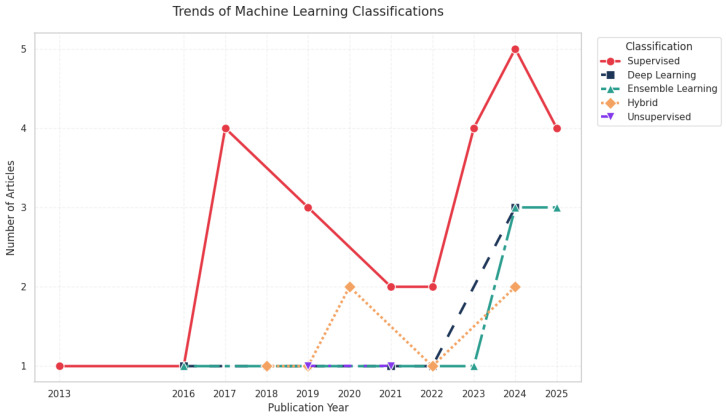
Distribution of AI methods used over the years.

The retained empirical literature employed a range of data-driven ML techniques, with supervised learning constituting the dominant methodological class. Figure 4 shows that most studies used supervised learning algorithms [90] to build predictive models or classifiers from labeled data, and cases in which unsupervised learning methods were used were also observed [91,92,93]. Within the supervised learning category, decision tree-based methods were the most frequently used. Frequently used approaches included artificial neural networks, random forests, support vector machines, gradient-boosting methods, and other classifiers or regressors adapted to process, formulation, or comparability datasets. Unsupervised approaches such as clustering and dimensionality-reduction methods [89,94,95] were also identified, particularly in analytical characterization and assessment of high-dimensional similarity. Importantly, the revised synthesis does not classify decision-analytic frameworks such as Markov models, semi-Markov models, or budget-impact models as ML. However, some studies [96,97,98,99,100] used Markov models for the optimization of ML models. Those approaches may be relevant for biosimilar policy or adoption, but do not learn from empirical data in the same sense as the data-driven algorithms included in this review. The resulting method taxonomy is therefore narrower than in the previous version but also more conceptually coherent.

Among these, ANNs were particularly common [101,102,103,104]. Several articles implemented neural network models, including deep learning architectures in some cases, to capture complex nonlinear relationships or even using image processing [105,106]. For example, multilayer forward-propagation neural networks [101,103] or more specialized deep learning models were used to predict results such as protein stability [105,107] or to model manufacturing processes with many interacting parameters [102]. In addition to ANNs, other commonly reported supervised ML techniques include tree-based ensemble methods [108]. In particular, Decision Trees (DTs) [89,95,96,97,98,99,100,109,110,111,112,113,114,115,116,117,118,119,120,121,122] are widely utilized due to their simplicity and inherent interpretability, which are crucial for regulatory and clinical validation, and similarly for RFs [92,104,108,123,124,125] and Gradient-Boosting Decision Trees (e.g., XGBoost) [117,120,121,126,127]. These algorithms are popular for their robustness and interpretability in assessing the importance of characteristics and have been applied in contexts such as feature selection for analytical characterization data and predictive modeling of process results or clinical responses. SVMs have also been used in various studies [123,124,125,128], often for classification tasks (e.g., classification of experimental outcomes or patient categories based on multiple input variables). Classical AI regression models (sometimes enhanced with regularization or nonlinear kernels) have been used in studies that seek to predict numerical outcomes such as binding affinities [88,94] or process performance metrics [109,113,114,115,116,118,119,129].

Some studies have used Bayesian approaches [130] or other advanced optimizers [97,98,99,100,110,111,112,122]; for example, a study used Bayesian optimization algorithms to efficiently search the formulation design space for a stable formulation of a biosimilar, illustrating the use of ML in the context of experimental design [97].

### 3.3. Biosimilar Stages Addressed by AI Applications

**Figure 5:**
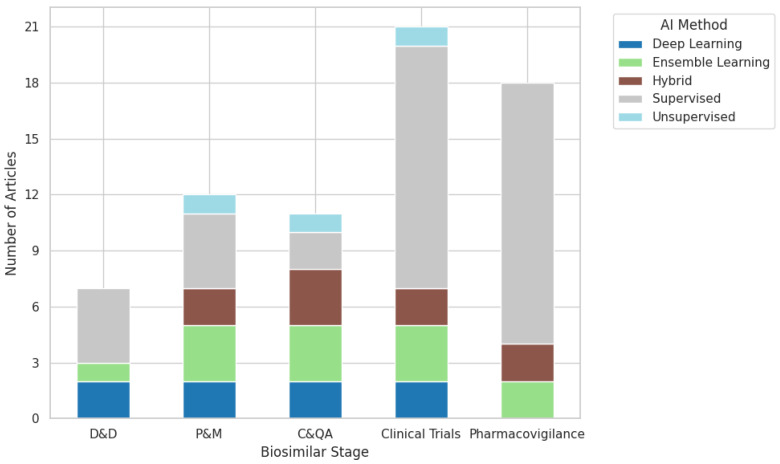
Stacked bar chart of the AI methods most frequently used in each stage of biosimilar development.

**Figure 6:**
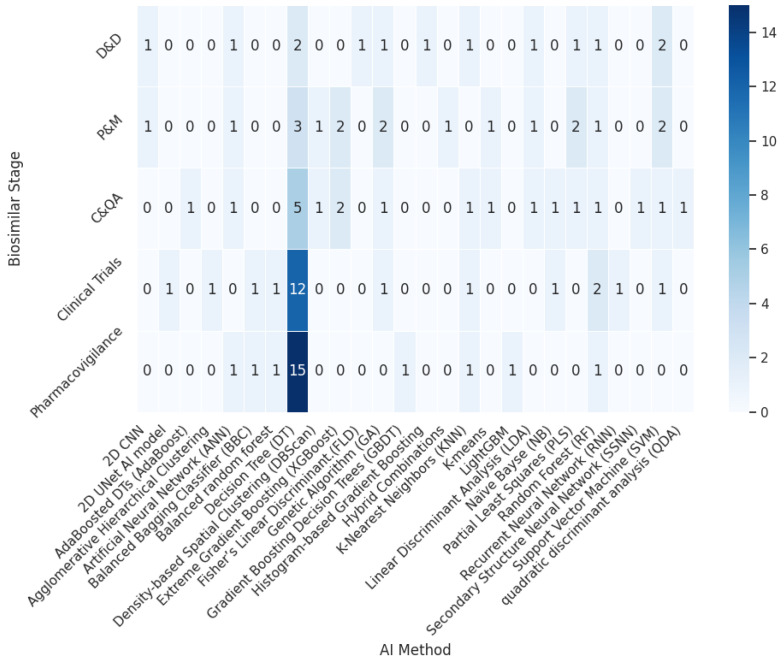
Heatmap of AI Methods by Biosimilar Stage.

An important aspect of this review is understanding which stages of the biosimilars development lifecycle has benefited from ML or AI solutions. Figure 5 and Figure 6 show that supervised methods are the most widely used in all stages of biosimilar development, with decision tree-based approaches the most prevalent. The most mature applications were observed in production/manufacturing and in analytical characterization/comparability, where data-rich environments enabled predictive modeling, multivariate similarity assessment, process optimization, and real-time quality-support strategies. By contrast, the empirical applications of ML in clinical evaluation and pharmacovigilance were fewer and more heterogeneous. These areas remain promising, but the biosimilar-specific evidence available is still comparatively limited. As a result, the revised synthesis supports a more differentiated interpretation of methodological maturity throughout the lifecycle rather than a uniform claim of broad AI penetration.

Similarly, unsupervised methods have appeared from the production stage through clinical trials, although their presence in these stages remains limited. Finally, the emergence of hybrid or mixed approaches can also be observed, which have contributed to improved model performance at several stages of biosimilar development.

#### 3.3.1. Design and Development (D&D)

A smaller number of studies addressed the initial phase of biosimilar development. At this stage, ML was used to facilitate the design and formulation of biosimilar [19,90]. The researchers applied ML models to predict the structural attributes or stability of a biosimilar candidate based on its amino acid sequence or formulation conditions [123]. Some cases were observed in which in silico modeling combined with ML was used to select and optimize protein engineering decisions, similar to the use of predictive algorithms to determine which protein variant might best fit the function of the reference product [105,128]. Another common application was in formulation development, where algorithms such as Bayesian optimization or neural network regressors helped explore the complex multivariate space of formulation components (buffers, excipients, etc.) to maintain product stability and similarity [19]. In this way, ML can reduce the number of laboratory experiments required, focusing efforts on the most promising formulation conditions [94,102,105]. In general, during the development stage, ML served as a decision support tool, guiding scientists in choosing designs with a higher probability of success or biosimilar to the original [107].

#### 3.3.2. Production and Manufacturing (P&M)

This stage was heavily featured in the literature, where some studies focused on bioprocess manufacturing processes and optimization of biosimilars [89,95,102,126,127]. Biological manufacturing (cell culture processes, purification, etc.) generates large amounts of process data (sensor readings, yields, quality attributes), and ML has proven valuable for extracting insights from these data [102]. The reviewed articles demonstrated the use of ML to model and optimize both initial processes (such as predicting how changes in fermentation conditions affect yield or product quality) and final processes (such as optimizing chromatography or filtration steps) [89,102,105,126]. Techniques such as ANNs and regression models were trained on historical process data to predict outcomes such as titer (protein yield) or impurity levels, enabling predictive control [89,102]. Some studies used ML for fault detection and process monitoring, where algorithms learned normal operating ranges and could detect anomalies that could lead to batch failures [91,102,126]. Others implemented multi-objective optimization, balancing factors such as maximizing throughput and minimizing production time, or ensuring that critical quality attributes remained within specifications [105]. The benefit of ML in manufacturing lies in improved efficiency and consistency: for example, a study demonstrated that ML-based optimization could increase production throughput by adjusting certain parameters in silico before laboratory verification [92]. Another example involved ML-enhanced Quality by Design frameworks, where the design of experiment data was incorporated into ML models to better understand how input parameters affect critical results [94,105]. In summary, the biosimilar production phase is a fertile field for ML, and the reviewed studies show that data-driven modeling can contribute significantly to process optimization, scaling decisions, and maintaining product equivalence during manufacturing.

#### 3.3.3. Characterization and Quality Control (C&QA)

A substantial portion of the studies (approximately one-quarter) fell into this category, which is fundamental to establish biosimilarity [62,91,126]. Analytical characterization generates high-dimensional data (from techniques such as chromatography, mass spectrometry, spectroscopy, bioassays, etc.) and ML methods have been used to interpret these complex datasets [7,91]. In our review, we found examples of the use of ML to more rigorously compare the biosimilar with the reference product [62,91,126,131]. In this case, algorithms can detect subtle patterns in spectroscopic data that distinguish batches or, conversely, confirm that there are no significant differences exist [89,91,97]. A study used a combination of principal component analysis and clustering in NMR (nuclear magnetic resonance) spectra to effectively characterize a biosimilar against its reference, demonstrating a quantitative assessment of “molecular similarity” using ML [123]. Other studies applied classification algorithms to predict whether a given sample met similarity criteria based on multiple attributes [91]. There have also been cases where ML has contributed to development, for example, by using neural networks to model and correct systematic biases in an analytical method, thus improving its precision in measuring biosimilarity [123]. Another interesting application has been the use of computer vision and pattern recognition in image data (such as two-dimensional electrophoresis gels or micrographs) to ensure consistent product quality [91]. In quality control, some studies have integrated ML for real-time analysis, whereby in real-time release testing concepts, an ML model predicts product quality attributes from process data, potentially reducing the need for extensive laboratory testing [9]. Together, these studies demonstrate that ML can improve the characterization stage by processing complex data and providing multidimensional comparisons that are more sensitive than traditional univariate analyses [131]. This helps biosimilar developers and regulatory bodies make more informed decisions regarding the similarity and release of products [7].

#### 3.3.4. Clinical Trials (Efficacy and Safety)

Although a considerable number of studies addressed the clinical or preclinical trial phases, this stage did not have the highest number of publications. Nevertheless, these articles demonstrated the important role of ML in assessing or predicting clinical outcomes for biosimilars [132]. In one example, an ML model was used to predict clinical efficacy endpoints (such as patient response rates) from baseline trial data or actual patient characteristics, which could be useful for trial design or to identify which subpopulations might respond differently to the biosimilar compared to the reference drug [108]. Another study focused on predicting immunogenicity: molecular and patient data were used to predict the likelihood of anti-drug antibody formation when switching to a biosimilar [133]. Such predictions could be valuable for the design of immunogenicity monitoring strategies or trial inclusion criteria [4]. In addition, we found instances of ML being applied to pharmacokinetic/pharmacodynamic (PC/PD) modeling [103,132]. A nonlinear mixed-effects model optimized using AI was used to compare the pharmacokinetic profiles of a biosimilar and its reference drug [108]. In general, while direct use of AI in the conduct of clinical trials is still in its early stages, these studies indicate a growing interest in using it to analyze clinical data more deeply, optimize trial efficiency, or improve post hoc analyses [133]. It should be noted that, because clinical datasets are typically smaller and noisier (since clinical outcomes involve numerous human confounders), the adoption of AI in this area has been cautious [4]. The limited number of studies in this category suggests that the application of AI in the clinical evaluation of biosimilars is still a developing field that will likely expand as more data become available (from completed trials and use in the real-world) [132].

#### 3.3.5. Pharmacovigilance

Some studies (the largest subset in our categorization) addressed the post-approval phase, using ML for pharmacovigilance and analysis of real-world evidence of biosimilars [2,7,108]. Pharmacovigilance involves monitoring adverse events and usage patterns once a drug is on the market, and ML can be invaluable in the analysis of large healthcare datasets for signals [86,114]. In our review, a prominent example used ML in pharmacovigilance databases to detect rare adverse events associated with biosimilars, essentially comparing the safety profiles of biosimilars and original biologics across thousands of reported events [108]. Techniques such as disproportionate analysis can be enhanced with ML classifiers to optimize signal detection (distinguishing genuine safety signals from noise) [2].

Furthermore, AI has been suggested as a tool for forecasting biosimilar adoption and utilization trends in various markets by analyzing prescription data and factors in the healthcare system [108]. We observe that while the applications of ML pharmacovigilance for biosimilars are currently limited, they are highly relevant. Importantly, long-horizon transition models, cost-effectiveness simulations, and payer-oriented adoption analyses were excluded from this section unless they incorporated a genuine trained learning component. This distinction yields a more conservative but scientifically defensible picture of the current biosimilar pharmacovigilance landscape of ML.

As more biosimilars enter the market, the volume of post-marketing data increases, making it easier to analyze using AI [99,115]. These initial studies demonstrate the feasibility of using ML to ensure the continued safety and efficacy of biosimilars, by rapidly detecting potential problems when switching patients or identifying immunogenic reaction patterns that might not be apparent with traditional methods [87,104,108]. This is a crucial area where we anticipate that more research will be conducted, since regulatory agencies have also begun to explore AI tools to monitor the safety of biological products (the FDA, for example, has introduced an AI-based system to help assess related risks immunogenetic in biosimilars [13]) [76].

Therefore, ML applications span the entire lifecycle of biosimilar products [19]. Most of the studies analyzed focus on the manufacturing and characterization stages (the middle-phase of the process), where data is abundant and the benefits (process efficiency, quality assurance) are immediately tangible [131]. Although present in the early stages of design and the later stages phases of clinical trials/pharmacovigilance, they are less common, likely reflecting both the novelty of these applications and the greater challenges in those areas (e.g., data scarcity or regulatory barriers) [7,118,119]. Nevertheless, even at these stages, we observed pioneering examples that indicate that the influence of ML is growing [19]. The multidisciplinary nature of these efforts, involving chemical engineers, data scientists, pharmacologists, and clinicians, underscores that the successful integration of ML in biosimilar development requires collaboration in different fields [131].

## 4. Discussion

### Main Findings

This systematic review shows that ML has begun to establish a meaningful role in biosimilar research, but its maturity is highly stage-dependent. A core set of 44 studies was found to have applied ML methods to address various challenges in biosimilar development. A key finding is that manufacturing/process optimization and analytical characterization are the areas where ML has been applied most intensively to date [123]. In contrast, clinical and commercialization applications [31] of ML for biosimilars, although emerging, were less common in the literature, suggesting that these areas are still developing. Once broader studies related to AI and HTA/economic decision models are separated from the core synthesis, the strongest evidence is concentrated in manufacturing/CMC and analytical comparability. This is expected since these stages generate structured, high-dimensional, and technically rich datasets that are well suited for supervised and multivariate modeling.

However, empirical biosimilar-specific ML applications in clinical prediction and pharmacovigilance remain relatively sparse. Their lower representation should not be interpreted as lack of relevance, but rather as an indication that these areas are still developing and face additional constraints related to sample size, data access, clinical heterogeneity, and regulatory interpretability.

The principal scientific contribution of this review therefore lies in clarifying where genuine empirical ML evidence currently exists in biosimilars, rather than in treating all computational or decision-support approaches as part of a single undifferentiated AI field.

Implications for biosimilar development: The increasing integration of algorithmic learning (AL) into biosimilar R&D has several important implications [64]. First, it represents a shift towards more efficient and data-driven development processes [105]. Traditional biosimilar development can be slow and expensive, for example, empirically determining the optimal manufacturing process or demonstrating biosimilarity through exhaustive analytical and clinical tests [131]. AI offers a way to streamline these efforts by learning from past data and guiding decision-making [81]. Our findings show that in manufacturing, AI models have successfully optimized conditions that improve yields or product quality, potentially reducing development time and costs [102,105]. In characterization, AI can improve the sensitivity and scope of comparisons between a biosimilar and its reference product, which can increase confidence in demonstrating similarity and possibly reduce the burden of certain laboratory tests if regulatory bodies accept validated models [131]. These advances, taken together, could reduce barriers to biosimilar development, which means that more biosimilar products could reach the market sooner and at a lower cost [81]. This is a crucial benefit, as biosimilars are key to expanding patient access to biologic therapies by offering more affordable alternatives [105]. If ML can help bring biosimilars to market more efficiently while ensuring high quality, it directly supports healthcare systems by saving costs and increasing the availability of treatments [131].

Furthermore, the application of ML in post-marketing surveillance (although currently limited) points to future where real-world evidence continuously guides the use of biosimilars [2]. Rapid detection of adverse event signals or efficacy issues using AI enables more agile responses (whether manufacturers adjust processes or physicians adapt usage guidelines) [102,134]. This proactive monitoring could improve the overall safety profile of biosimilars and maintain trust among healthcare professionals and patients [108]. In the long term, the widespread use of these tools could even become part of regulatory expectations, requiring companies that market biosimilars to also employ validated AI systems to continuously monitor the performance of their products on the market [2].

During selection, we identified a secondary body of literature addressing broader topics related to AI, as well as HTA, budget-impact, cost-effectiveness, switching scenarios, and market-access models in biosimilars. These studies are relevant to the wider biosimilar ecosystem because they inform policy, payer behavior, adoption dynamics, and long-term treatment pathways. However, they do not constitute empirical ML evidence unless they include an actual trained learning component. Their separation from the core synthesis is, therefore, methodological rather than dismissive. In other words, these studies remain valuable for contextual interpretation, but they should not shape the main counts, figure distributions, or conclusions regarding empirical ML maturity in biosimilars. This distinction substantially improves the conceptual precision of the manuscript and directly addresses the reviewer’s concern about scope inflation.

## 5. Comparison with Other Areas

The pattern observed in biosimilars reflects broader trends in the biopharmaceutical industry. ML is increasingly being adopted for the development of biologics in general, not just biosimilars, but also novel biologics, for tasks such as protein design, manufacturing optimization, and clinical trial analysis [81]. Biosimilars present a particularly compelling case for ML, as the goal is to match the profile of an existing product as closely as possible; this creates a clear objective for optimization algorithms (e.g., minimizing differences from the reference product). In this respect, some challenges in biosimilar development align well with the ML optimization paradigms. Our review confirms that researchers are using techniques similar to those in related fields such as bioprocess engineering and chemometrics, but applying them with the specific intention of meeting biosimilarity criteria. For example, the use of ML to ensure compliance with Quality by Design principles is not unique to biosimilars, but in this context, the tolerance for deviation is lower, making the accuracy of ML even more valuable. Furthermore, previous reviews specifically on ML for biosimilar safety monitoring highlight that combining the complexity of biologics with modern analytics can generate new insights, an idea that our general findings reinforce [63]. In summary, the field of biosimilars appears to be following and amplifying the general trajectory of AI adoption in the pharmaceutical industry, confirming that these tools are versatile and beneficial for solving biopharmaceutical problems.

## 6. Challenges and Future Prospects

Despite promising results, significant challenges remain for the full integration of ML into biosimilar development. One major challenge is the need for large and high-quality datasets. Training robust ML models, especially deep learning models, typically requires a large amount of data. At some stages of biosimilar development, data may be limited: for example, sample sizes in clinical trials are relatively small, and even single-product manufacturing datasets may not be very extensive (compared to, for example, reference datasets for image recognition). This data scarcity can limit model performance or lead to overfitting. To address this issue, future research could focus on techniques such as data enhancement, transfer learning (perhaps leveraging data from related biologics), or federated learning across companies or institutions to create larger collaborative datasets. Another challenge is interpretability and trust. Regulatory agencies and the pharmaceutical industry must have confidence in ML-based decisions. Black-box models (such as certain deep neural networks) can generate skepticism unless they are interpretable or can be rigorously validated. We observed that many studies opted for more interpretable methods (e.g., decision trees, simpler regression models) when the context required clarity, such as in process control or release decisions. In the future, the development of explainable AI techniques, tailored to clinical and bioprocessed data, will be valuable as AI models can not only provide predictions but also insights into the reasons behind them; this could accelerate the regulatory acceptance of AI tools. In fact, the regulatory landscape is evolving; initiatives by regulatory bodies (e.g., the FDA’s exploration of AI tools to assess protein aggregation and risk of immunogenicity) indicate a growing openness to incorporate ML into biologics monitoring. However, harmonization with regulatory standards remains a hurdle; guidelines will need to be updated on how to validate and document ML models as part of biosimilar approval applications.

Another challenge is the lack of interdisciplinary knowledge among many healthcare professionals on the successful use of drugs in biosimilars. This requires the collaboration of data scientists and experts in the field of medications, such as pharmacists. They possess the necessary and indispensable knowledge in chemistry, biology, microbiology, pharmaceutical technology, biopharmaceutics, pharmacokinetics, pharmacology, pharmacotherapy, pharmacogenomics, and toxicology. They also have a deep understanding of the regulatory aspects related to the medications they develop. We suspect that some areas (such as clinical trial design) have experienced slower adoption, in part because both communities of scientists need to converge. As more junior professionals with data science skills enter the biopharmaceutical sector, we anticipate a cultural shift where such collaborations become more common, thereby driving greater innovation. The reviewed studies already show some interdisciplinary teams and this trend is expected to continue.

## 7. Conclusions

The main contribution of this article is the distinction between genuine ML applications and adjacent AI-related or decision-analytic literature in the biosimilar field. Under this stricter definition, the evidence base is smaller, but scientifically more coherent. The revised synthesis indicates that the most mature empirical ML applications are currently concentrated in manufacturing and analytical comparability, where data-rich environments allow meaningful gains in process optimization, multidimensional similarity assessment, and quality-support analytics. Clinical prediction and pharmacovigilance remain important, but relatively less developed areas for biosimilar-specific ML. These findings suggest that the future of ML in biosimilars will depend not only on methodological innovation but also on improved data access, transparent validation practices, and closer collaboration between biopharmaceutical scientists, data scientists, clinicians, and regulators.

This article also reveals that ML is gaining significant momentum in the biosimilar sector, providing novel optimization paths. This growing trajectory suggests a shift toward more data-driven methodologies, even as adoption levels remain diverse across the distinct phases of research. The evidence available to date shows clear benefits: it improves efficiency in development and manufacturing, increases the rigor of analytical comparisons, and opens new possibilities in clinical data analysis and post-marketing surveillance. These advances support the larger mission of biosimilars: providing affordable, high-quality biologic therapies to patients. Furthermore, ML that improve safety and efficacy could be improved by analyzing complex data that would be difficult to interpret using traditional methods, thus maintaining confidence in the use of biosimilars. The intersection of AI and biosimilars is still evolving and has significant growth potential. As data resources expand and technology advances, we anticipate greater integration of intelligent systems at every stage of the biosimilar product lifecycle. Continued collaboration between computational scientists, biopharmaceutical experts, and regulatory bodies will be critical to fully harness the potential of ML; however, the trajectory is already set. The advances discussed in this article represent steps towards a future where biosimilar development is faster, smarter, and even more reliable, ultimately benefiting healthcare systems and patients worldwide.

## 8. Limitations of  Research

This research has many limitations. First, the search was restricted to Scopus, PubMed and Web of Science and to English-language publications, which may have led to the omission of some relevant studies. Second, the field remains relatively small (44 articles), is modest for robust quantitative analysis, and the final core empirical ML corpus does not support meta-analysis because of methodological and outcome heterogeneity. Third, the distinction between the empirical evidence for the core of ML and the contextual computational literature, although methodologically necessary, involves some degree of judgment during full-text evaluation. To reduce ambiguity, this issue should be addressed through explicit operational definitions and consensus-based screening. Finally, the refined evidence base should be interpreted as a map of methodological maturity rather than as a quantitative performance ranking of algorithms.

There is a degree of subjectivity in this process. Nevertheless, we believe that the chosen categories (five key stages and broad classes of ML methods) adequately reflect the landscape. Finally, our citation analysis is only up to date with the search; the number of citations will have increased since then, particularly for very recent articles, but the qualitative observation that older studies have a greater influence likely still holds true.

## 9. Future Research Opportunities

- Applying advanced ML techniques (such as reinforcement learning or more sophisticated deep learning models) to biosimilar problems that have not yet been fully explored, such as adaptive clinical trial simulations or real-time release tests with IoT data integration, is beginning to see some conceptual work in these directions, but practical implementations are still few.
- Expanding the scope of data used by the ML, for example, by incorporating omics data (genomics, cell line proteomics) to improve the development of cell lines for biosimilars, or using ML to integrate multi-attribute analytical data for a holistic similarity assessment.
- Evaluating the generalizability of ML models across products: an interesting question is whether a model trained on data from one biosimilar process could be transferred or adapted to another product with minimal retraining, providing insight into the underlying principles that ML captures; this could make development easier in the future.

## Figures and Tables

**Table 1:** Summary of search strategies in electronic databases.

Database	Advanced Search
Scopus	TITLE-ABS-KEY (biosimilar* OR “follow-on biologic*”) AND TITLE-ABS-KEY (development OR design OR formulation OR optimization OR manufacturing OR production OR “process optimization” OR bioprocess* OR “quality control” OR comparability OR characterization OR analytics OR “clinical trial*” OR efficacy OR safety OR immunogenicity)
WoS	TS = (biosimilar* OR “follow-on biologic*”) AND (development OR design OR formulation OR optimization OR manufacturing OR production OR “process optimization” OR bioprocess* OR “quality control” OR comparability OR characterization OR analytics OR “clinical trial*” OR efficacy OR safety OR immunogenicity)
PubMed	(“Biosimilar Pharmaceuticals”[Mesh] OR biosimilar*[tiab] OR “follow-on biologic*”[tiab]) AND (“Drug Development”[Mesh] OR “Biological Products”[Mesh] OR development[tiab] OR design[tiab] OR formulation[tiab] OR optimization[tiab] OR manufacturing[tiab] OR production[tiab] OR “process optimization”[tiab] OR bioprocess*[tiab] OR “quality control”[tiab] OR comparability[tiab] OR characterization[tiab] OR analytics[tiab] OR “clinical trial*”[tiab] OR efficacy[tiab] OR safety[tiab] OR immunogenicity[tiab])

* The asterisk (*) is a truncation wildcard for word variations.

## Data Availability

Data is contained within the article or Supplementary Material.

## Supplementary Materials

The following supporting information can be downloaded at: https://www.mdpi.com/article/10.3390/ph19050745/s1, S1: Python script (.py) (3.12.3)—Script of python source code used for automated record cleaning, deduplication, and metadata extraction. The script generating statistical plots and bibliometric trends. S2 Results/: Folder—High-resolution visualizations (distribution by development stage, algorithm frequency, etc.). S3: text (.csv)—Original search results exported as .csv or .bib from Scopus and Web of Science (WoS), and join in one file .csv. S4: Excel (.xlsx)—The final curated Review Matrix including paper classifications and technical justifications.

## Appendix A. Review Matrix

**Table A1:** Review Matrix: AI and ML in Biosimilars.

Paper	Core Algorithms/Models	Biosimilar Development Stage	Why
Alam et al., 2024 [105]	2D CNN	Production	Interprets CNN outputs using SHAP and Bayesian optimization to match biosimilar CQAs to the reference molecule.
Aliyev et al., 2019 [96]	Decision Tree (DT)	Clinical/HTA	Uses decision trees to demonstrate higher effectiveness and cost-efficiency of biosimilars.
Alizadeh et al., 2024 [97]	Decision Tree (DT)	Preclinical/CMC	Establishes cost-effective dosing strategies and long-term biologic maintenance through clinical-economic modeling.
Blanken et al., 2018 [129]	Decision Tree (DT)	Econ/HTA	Validates the economic efficiency of biosimilar entry in respiratory health through probabilistic sensitivity analysis.
Borget et al., 2025 [135]	Decision Tree (DT)	Clinical/HTA	Implements decision-tree modeling to support clinical decision-making and cost-effectiveness in reproductive health.
Brühlmann et al., 2017 [94]	Decision Tree (DT)	Production	Automates culture supplement selection via multivariate analysis and decision trees to optimize protein glycosylation.
Castro-Corredor et al., 2023 [108]	Balanced Bagging Classifier (BBC); Balanced random forest	Clinical/RWE	Applies ML to real-world data to predict therapy discontinuation and clinical response in biosimilar users.
Catt et al., 2019 [109]	Decision Tree (DT)	Clinical/HTA	Models risk-benefit ratios and economic impacts of switching to infliximab biosimilars under clinical uncertainty.
Chakravarty et al., 2021 [103]	Recurrent Neural Network (RNN)	Preclinical/CMC	Refines physiologically-based pharmacokinetic models using ML to predict biosimilar exposure and systemic distribution.
Dilokthornsakul et al., 2023 [110]	Decision Tree (DT)	Clinical/HTA	Projects long-term cost-utility and clinical response quality for biosimilars compared to standard biological therapy.
Fenu et al., 2022 [111]	Decision Tree (DT)	Clinical/HTA	Predicts clinical remission rates and sustained therapeutic benefits for biosimilars using hybrid transition models.
Fischer et al., 2025 [112]	Decision Tree (DT)	Clinical/HTA	Analyzes the impact of biosimilar pricing on treatment sequences and health-economic scenarios for anti-TNF therapies.
Gangwar et al., 2024 [92]	Random Forest (RF); Hybrid Combinations	Production/CMC	Evaluates the impact of media components on critical charge variants using gradient boosting to ensure biosimilarity.
Germeni et al., 2024 [113]	Decision Tree (DT)	Clinical/HTA	Quantifies the economic and clinical consequences of switching or discontinuing biosimilar anti-C5 therapies.
Hamla et al., 2022 [128]	Partial Least Squares Regression (PLSR); Support Vector Machine Regression (SVMR)	CMC/Dev	Employs non-linear SVR to predict glycosylation attributes as a high-throughput alternative for analytical similarity.
Hernandez et al., 2020 [98]	Decision Tree (DT)	Post-approval/HTA	An economic model using a hybrid decision tree is developed to evaluate biosimilar pricing and market access scenarios.
Hombalimath et al. [101]	ANN (Neural Network)	Production	Optimizes enzymatic bioprocess yield and consistency through empirical neural network modeling.
Hou et al., 2025 [104]	Random Forest (RF); Gradient Boosting Decision Trees (GBDT); Artificial Neural Network (ANN); LightGBM; K-Nearest Neighbors (KNN)	Post-approval/Clinical	Predicts clinical flare risk following non-medical switching to infliximab biosimilars using automated ML.
Hudnik et al., 2023 [90]	Partial Least Squares (PLS)	Market	Forecasts market uptake and budget impacts of new biosimilar entries through data-driven sensitivity analysis.
Jalali et al., 2013 [134]	Genetic Algorithm (GA); Linear Discriminant Analysis (LDA); Support Vector Machine (SVM)	CMC/Characterization	Applied multiple ML classifiers to evaluate the analytical similarity and structural integrity of therapeutic proteins.
Karagianni et al., 2019 [93]	Agglomerative Hierarchical Clustering; Random Forest (RF)	Preclinical	Identifies molecular efficacy signatures via RF to demonstrate similarity in mechanisms of action at the systems level.
Kim et al., 2016 [123]	K-Nearest Neighbors (KNN); Support Vector Machine (SVM); Linear Discriminant Analysis (LDA); quadratic discriminant analysis (QDA); Naïve Bayse (NB); Decision Tree (DT); Random Forest (RF); AdaBoosted DTs (AdaBoost)	CMC/Characterization	Validates multiple ML classifiers for robust analytical comparability and decision-making in bioprocess development.
Lehmann et al., 2024 [114]	Decision Tree (DT)	Clinical/HTA	Develops validated models to refine clinical assumptions and dose distribution for follicle-stimulating biosimilars.
Olfatifar et al., 2022 [115]	Decision Tree (DT); microsimulation (MS) approach	Post-approval/HTA	Projects quality-adjusted life years (QALYs) and economic impacts for biosimilars in emerging healthcare markets.
Orsini et al., 2024 [106]	2D UNet (AI)	Clinical/Monitoring	Applies deep learning for precise morphological monitoring of skin responses to biological and biosimilar therapies.
Osiri et al., 2021 [116]	Decision Tree (DT)	Clinical/HTA	Evaluates budget impact and resource allocation over multi-year horizons following the introduction of targeted biosimilars.
Pathak et al., 2022 [102]	ANN (Neural Network)	CMC/Development	Predicts mixed-mode chromatographic performance to establish operational design spaces for teriparatide biosimilars.
Petryszyn et al. [99]	Decision Tree (DT)	Clinical/HTA	Ranks the cost-effectiveness of therapeutic sequences under varying biosimilar pricing and discount scenarios.
Prabha et al., 2024 [124]	Naïve Bayse (NB); Random Forest (RF); K-Nearest Neighbors (KNN); Support Vector Machine (SVM)	CMC/Characterization	Classifies structural fidelity and molecular identity of inhibitors using ML-based chemical fingerprints.
Shatat et al., 2021 [91]	K-means; DBScan	Production	Employs unsupervised clustering of peptide mapping data to ensure identity and high-resolution biosimilarity.
Shrivastava et al., 2025 [127]	Extreme Gradient Boosting (XGBoost)	Production/Analytics	Quantifies glycan similarity indices (GIs) using gradient boosting for automated real-time quality control.
Shrivastava, et al., 2024 [126]	Extreme Gradient Boosting (XGBoost)	Production	Applied gradient boosting algorithms to quantify glycan similarity indices (GIs) and ensure real-time quality control during biosimilar manufacturing.
Sklepari et al., 2016 [107]	Secondary Structure Neural Network (SSNN)	Characterization	Deconvolves complex spectral data using neural networks to confirm higher-order structural comparability in insulin.
Sokolov et al., 2017 [95]	Decision Tree (DT)	Econ/HTA	Models the dynamic adoption and health system utility associated with the transition to biosimilar-based care.
Sokolov et al., 2018 [89]	Decision Tree (DT); Partial Least Squares (PLS); Genetic Algorithm (GA)	Production	Systematically optimizes upstream bioprocesses to improve the yield and consistency of critical quality attributes.
Spahn et al., 2017 [130]	Genetic Algorithm (GA)	Development/Production	Uses genetic algorithms and Markov models to design cell lines that match the specific glycan profiles of the originator.
Sussell et al., 2022 [100]	Decision Tree (DT)	Clinical/HTA	Used decision-tree with Markov modeling optimization to evaluate the long-term cost-effectiveness and therapeutic value of switching to biosimilars in chronic care.
Tarallo et al., 2019 [117]	Decision Tree (DT)	Econ/HTA	Quantifies the economic impact and resource utilization shifts following the clinical switch to etanercept biosimilars.
Tjakra et al., 2025 [125]	Support Vector Machine (SVM); K-Nearest Neighbors (KNN); Histogram-based Gradient Boosting; Decision Tree (DT); Random Forest (RF); Fisher’s Linear Discriminant (FLD)	Preclinical/Formulation	Classifies drug diffusion through biological barriers via ML to evaluate bioequivalence in novel delivery systems.
Torre et al., 2024 [118]	Decision Tree (DT)	Econ/HTA	Estimates adherence-related cost savings and utility gains for new long-acting biosimilar insulin formulations.
Virani et al. 2024 [119]	Decision Tree (DT)	Clinical/HTA	Evaluates incremental cost-effectiveness ratios (ICERs) of biosimilars based on vision-related clinical outcomes.
Wang et al., 2017 [120]	Decision Tree (DT)	Econ/HTA	Analyzes market dynamics and price competition triggered by the introduction of filgrastim biosimilars.
Xue et al., 2019 [121]	Decision Tree (DT)	Econ/HTA	Compares the cost per live birth of originator vs. biosimilar follitropins in assisted reproduction through decision modeling.
Zhang et al., 2023 [122]	Decision Tree (DT)	Clinical/HTA	Maps health-related quality of life and efficacy trade-offs for biosimilars using Bayesian network meta-analysis.
