# Supplementary material for: Advances in Biosimilars: A Systematic Review of Machine Learning Applications
Source: Pharmaceuticals (Basel). 2026 May 8;19(5):745. doi: 10.3390/ph19050745 (PMC13209414; doi:10.3390/ph19050745)

PRISMA Flow Diagram for the Systematic Review

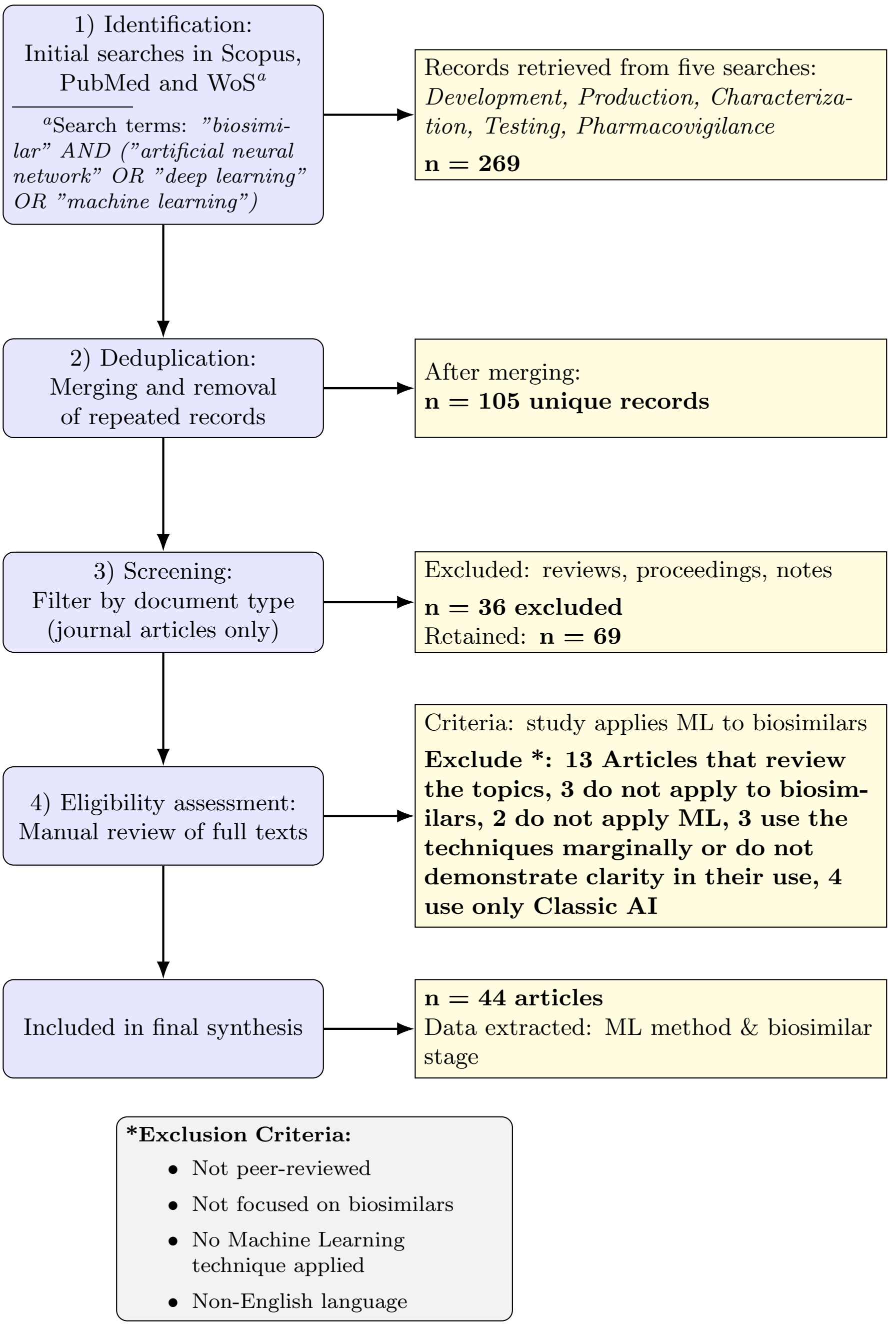

Supplement: Supplementary file 1 [file pharmaceuticals-19-00745-s001.zip › Results/methodology.pdf]
