# Supplementary figures and images for: Advances in Biosimilars: A Systematic Review of Machine Learning Applications
Source: Pharmaceuticals (Basel). 2026 May 8;19(5):745. doi: 10.3390/ph19050745 (PMC13209414; doi:10.3390/ph19050745)

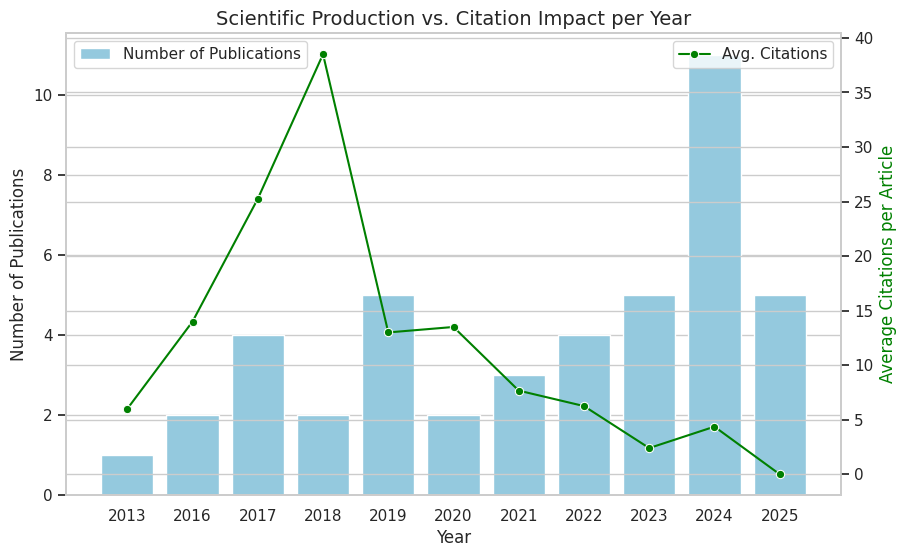

Supplement: Supplementary file 1 [file pharmaceuticals-19-00745-s001.zip › Results/ArticlesperYear.png]

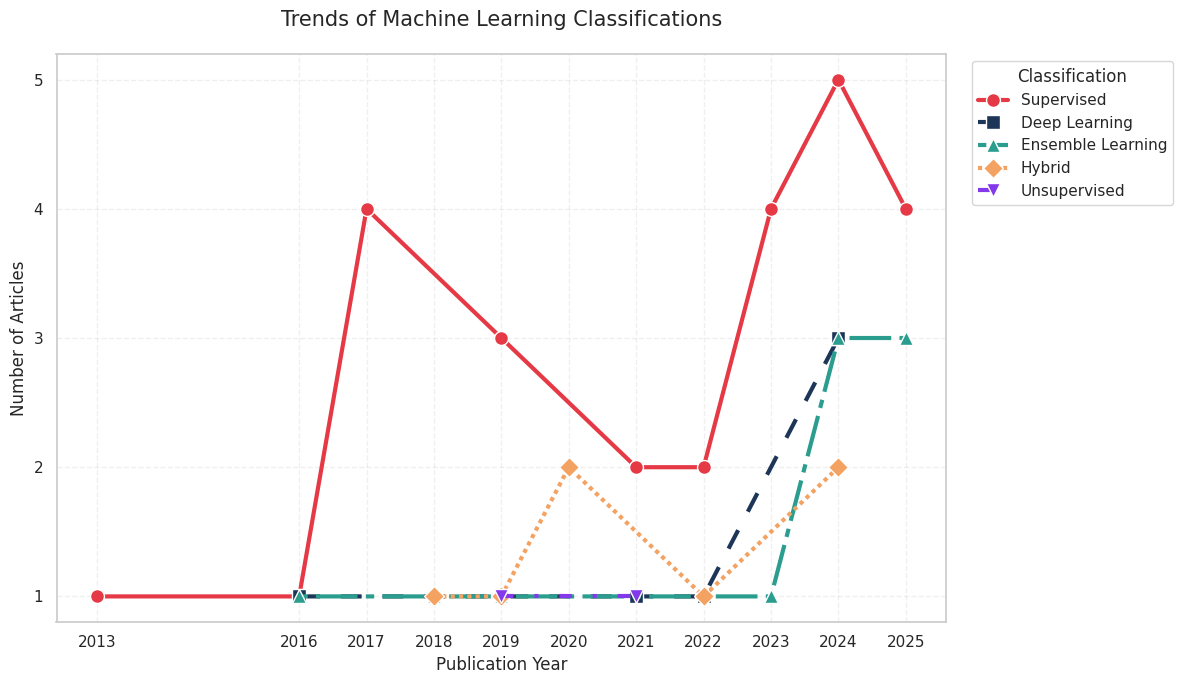

Supplement: Supplementary file 1 [file pharmaceuticals-19-00745-s001.zip › Results/MethodsPerYear.png]

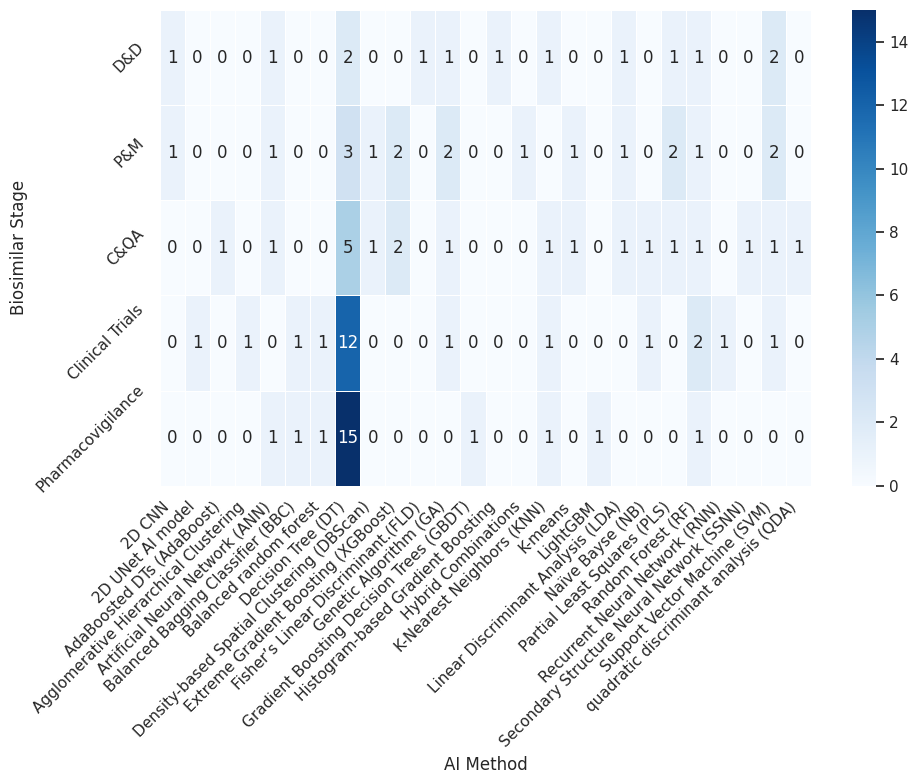

Supplement: Supplementary file 1 [file pharmaceuticals-19-00745-s001.zip › Results/Heatmapstage.png]

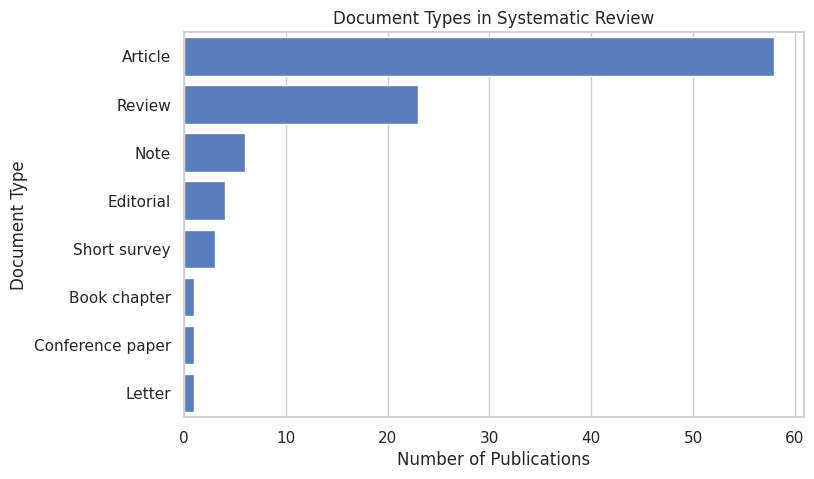

Supplement: Supplementary file 1 [file pharmaceuticals-19-00745-s001.zip › Results/TypeofArticle.png]

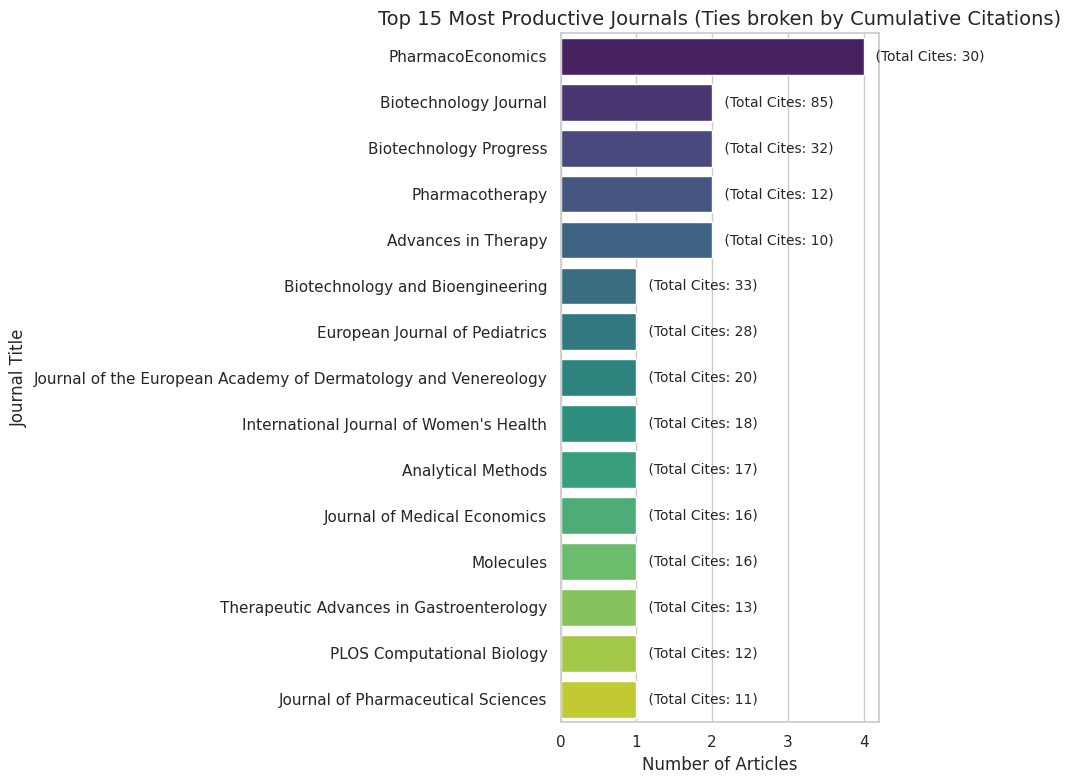

Supplement: Supplementary file 1 [file pharmaceuticals-19-00745-s001.zip › Results/top15.png]

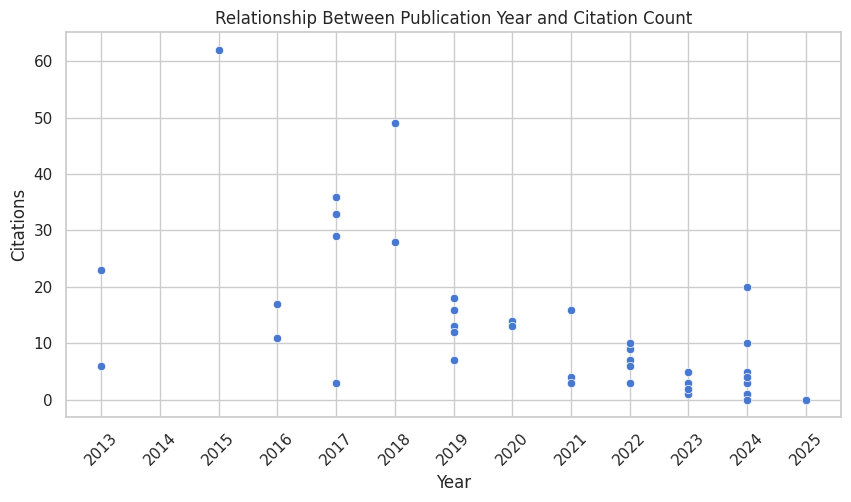

Supplement: Supplementary file 1 [file pharmaceuticals-19-00745-s001.zip › Results/citesperYear.png]

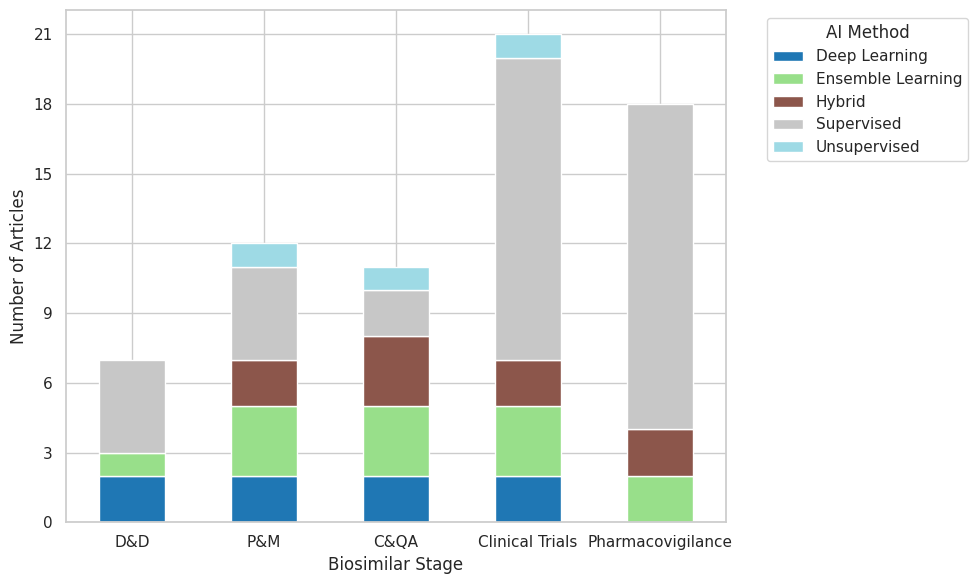

Supplement: Supplementary file 1 [file pharmaceuticals-19-00745-s001.zip › Results/methodStage.png]
